# Occurrence of Enteric Viruses in Multi-Component Salad Bowls

**DOI:** 10.3390/v18091000

**Published:** 2026-09-11

**Authors:** Mariana Alves Elois, Nadine Yeramian, Daniel Perez-Alonso, Rafael Dorighello Cadamuro, Antonio Valero-Díaz, Álvaro Cañete-Reyes, Henrique Borges da Silva, Gislaine Fongaro, David Rodríguez-Lázaro

**Affiliations:** 1 Laboratory of Applied Virology, Department of Microbiology, Immunology, and Parasitology, Federal University of Santa Catarina, Florianópolis 88035-972, Brazil; malves@ubu.es (M.A.E.); rdorighello@ubu.es (R.D.C.); hgrisard@gmail.com (H.B.d.S.); gislaine.fongaro@ufsc.br (G.F.); 2 Microbiology Area, University of Burgos, 09001 Burgos, Spain; nyeramian@ubu.es (N.Y.); dperez@ubu.es (D.P.-A.); acanete@ubu.es (Á.C.-R.); 3 Centre for Emerging Pathogens and Global Health, University of Burgos, 09001 Burgos, Spain; 4 Department of Food Science and Technology, Unidad de Investigación Competitiva de Zoonosis y Enfermedades Emergentes (ENZOEM), University of Córdoba, 14014 Cordoba, Spain; bt2vadia@uco.es

**Keywords:** foodborne viruses, ready-to-eat foods, multi-component meals, last-mile delivery, viral surveillance, food safety

## Abstract

The expansion of e-commerce and last-mile food delivery has created an increasingly relevant but understudied context for food safety surveillance. However, the occurrence of enteric viruses in ready-to-eat (RTE) foods within this distribution context remains largely unexplored. This study aimed to investigate the presence of enteric viruses in multi-component RTE salad bowls obtained through last-mile delivery. The study was designed to characterize the virological status of products received through this distribution pathway, without attributing any detected contamination specifically to the delivery process itself. A total of 30 salad samples were collected in two independent batches over a two-month interval via a third-party e-commerce platform and delivered directly to the laboratory. Virus concentration was performed using a method based on ISO 15216-1:2017, followed by RNA extraction and reverse transcription quantitative polymerase chain reaction (RT-qPCR) detection of norovirus (NoV) genogroups I (GI) and II (GII), hepatitis A virus (HAV), hepatitis E virus (HEV), human astrovirus (HAstV), and rotavirus (RV). HEV-target RT-qPCR signals were observed in 5 of 30 samples (16.7%); however, all positive signals corresponded to levels below the estimated limit of quantification (LOQ). No RT-qPCR amplification was observed for the other targeted viruses. This study provides initial occurrence data for enteric viruses in multi-component RTE meals obtained through last-mile delivery and supports the inclusion of this food category and emerging distribution pathways in foodborne virus surveillance.

## 1. Introduction

In the past decade, e-commerce has experienced steady and sustained growth. Accelerated by the COVID-19 pandemic, online retail expanded exponentially, increasing the demand for last-mile delivery services [1]. Last-mile food delivery refers to the final stage of the delivery process, in which food is transported from a food distribution center, restaurant, or supermarket to the consumer or, less commonly, to another business [2,3]. The delivery process involves ordering food online, typically facilitated by smartphone applications that allow users to select restaurants, menus, and prices. Orders and payments can be made directly by the customer on the establishment’s online platform or through a third-party e-commerce platform [4]. Deliveries may be carried out by the restaurant or catering service itself, or by the delivery service associated with the platform [5]. However, the occurrence of enteric viruses in foods obtained through last-mile delivery pathways remains poorly characterized. Existing research has mainly examined viral contamination in minimally processed products such as salads, fruits, vegetables, seafood, and some ready-to-eat (RTE) foods [6,7,8,9,10].

Viruses are recognized as important emerging foodborne pathogens. According to the 2024 European Union One Health Zoonoses Report, viruses were responsible for 677 foodborne outbreaks in the European Union (EU), representing 10.3% of all reported outbreaks, with norovirus (NoV) and other caliciviruses accounting for the highest number of outbreak-related human cases (14,297 cases) [11]. Enteric viruses comprise a diverse group of viruses transmitted predominantly through the fecal–oral route. Depending on the viral agent, infection may result in gastroenteritis or hepatitis, and infected individuals may shed large quantities of viral particles in feces [12]. Inadequate water treatment, combined with the high environmental stability of these viruses, allows them to remain infectious and perpetuate the cycle of food and water contamination [13,14]. Foodstuffs may become contaminated through direct contact with infected individuals during handling or processing, or indirectly via environmental sources such as inadequately treated irrigation water, improper use of organic waste as fertilizer, or direct contamination from livestock, wild animals, and birds [15]. Since fresh produce and many RTE foods are often consumed raw and without further washing or decontamination, they represent important transmission routes for human enteric viruses [9,16].

The Food and Agriculture Organization of the United Nations and the World Health Organization (FAO/WHO) have identified NoV and hepatitis A virus (HAV) in fresh and frozen produce and prepared or RTE foods as important virus–commodity combinations for food safety. However, other viruses are also relevant to food safety, as they can contaminate food and water and be transmitted to humans through these routes. This includes hepatitis E virus (HEV) [17], as well as rotavirus (RV), human astrovirus (HAstV), sapovirus, and human adenovirus (hAdV) [18].

Foodborne transmission of HAV through fresh and frozen produce has been repeatedly documented in Europe. Examples include an outbreak associated with semi-dried tomatoes in the Netherlands [19], a multinational Nordic outbreak linked to frozen strawberries [20], and a large European outbreak associated with frozen mixed berries that involved 1589 reported cases between 2013 and 2014 [21]. More recently, an HAV outbreak involving 24 patients in the Netherlands in 2024–2025 was linked to frozen blueberries [22]. Regarding the occurrence of enteric viruses in fresh produce and RTE foods, a survey of 911 RTE vegetable samples in Italy detected HAV and HEV RNA in 1.9% and 0.6% of samples, respectively [8]. Similarly, NoV and RV were detected in 6% and 0.4% of RTE packaged leafy green samples, respectively, in a Canadian retail survey [23]. In leafy green vegetables collected in Argentina, RV and HAstV genomes were detected in 5% and 32% of crop samples, respectively [24]. Additionally, NoV RNA has been detected in RTE salad vegetables [25], as well as in samples collected from lettuce production, processing, and retail environments [26], and in lettuce, watercress, and tomatoes [9]. These findings indicate that fresh produce and RTE foods can be contaminated by a range of enteric viruses and support the inclusion of multiple viral targets in surveillance studies.

This work assessed the occurrence of NoV genogroups I (GI) and II (GII), HAV, HEV, HAstV, and RV in multi-component RTE salad bowls purchased through a third-party e-commerce platform and analyzed after last-mile delivery. By evaluating the products as received by consumers, the study provides data on enteric virus occurrence in a food category and purchasing context that remain poorly characterized, without attempting to identify the stage at which contamination occurred or to attribute contamination specifically to the last-mile delivery process.

## 2. Materials and Methods

### 2.1. Sample Selection and Collection

A market survey was conducted in Burgos (Spain) to find restaurants and catering facilities that supply RTE multi-component salad bowls, often marketed as “Poke bowls”. Poke originated in Hawaiian cuisine and traditionally consists of raw, sliced or cubed, marinated fish or seafood served over rice [27]. However, the salads included in this study had more diverse compositions, containing vegetables (e.g., leafy greens) or carbohydrates (e.g., rice or pasta) as a base, accompanied by a protein component (e.g., cooked or smoked salmon, cooked chicken or ham) and additional ingredients (Table S1).

Salad bowls from three different restaurants (salad type) were evaluated (A, B and C). For each type, five independent samples were collected during the first sampling period, and five independent samples were collected during the second sampling period, resulting in 10 samples per salad type and 30 independent samples in total. Samples obtained during the two sampling periods (July and September) were considered independent physical samples and were not treated as technical replicates. All samples were acquired through orders on a third-party e-commerce platform and delivered directly to the laboratory facilities. In addition, the online menus of the three establishments were reviewed to determine whether pork-containing dishes were offered.

All samples were received in cardboard bowls with plastic lids and were transported without active refrigeration. Transport times ranged from approximately 10 to 30 min. Samples were obtained through a third-party delivery platform and transported by bicycle or car. Detailed sample-specific delivery conditions, weights, and temperatures are provided in Table S2.

Laboratory personnel were not blinded to sample identity during molecular analysis. Nevertheless, all samples were processed according to the same predefined analytical procedures and positivity criteria.

### 2.2. Physicochemical Analysis

Thirty independent salad bowls were collected across two sampling periods, with 15 samples collected per period and analyzed for physicochemical properties (pH, temperature, and water activity).

Upon last-mile delivery to the laboratory, the food sample packaging was opened aseptically, and the core temperature of the salad bowl was measured immediately by inserting a thermometer (Digital Thermometer SCALA^®^, GEFU GmbH, Eslohe, Germany) into the center of the bowl. A representative subsample of the composite food (25 g) was used for virological analysis. An additional 25 g subsample was used for the determination of pH (Handheld General Purpose Food and Dairy pH Meter, Hanna Instruments, Woonsocket, RI, USA) at 25 °C and water activity (AQUALAB 4TE, Decagon Devices, Inc., Pullman, WA, USA).

### 2.3. Virus Concentration and Nucleic Acid Extraction

Virus concentration was performed using a method based on International Organization for Standardization (ISO) 15216-1:2017, with minor modifications [28]. Briefly, twenty-five grams of each sample were weighed, transferred to sterile homogenization bags containing 40 mL of tris-glycine-beef extract (TGBE) buffer (Tris and glycine, SIGMA, Merck Life Science, Madrid, Spain; beef extract, Lab-Lemco powder, Oxoid, Madrid, Spain), and inoculated with 2.49 × 10^4^ Infective Units (IU) of Mengovirus (MgV) (vMC0, Spanish Type Culture Collection [CECT], Valencia, Spain) as a process control virus. Samples were agitated on an orbital shaker (Luckham R300, Cole-Parmer, Vernon Hills, IL, USA) for 20 min at room temperature. The recovered buffer was centrifuged at 8000 rpm for 30 min at 4 °C (Centrifuge 5810 R, Eppendorf SE, Hamburg, Germany), and the pH of the resulting supernatant was adjusted from 9.5 to 7.0 by the addition of 10 N hydrochloric acid (HCl, SIGMA, Merck Life Science, Madrid, Spain). Subsequently, 0.25 volumes of 5× polyethylene glycol (PEG) 8000/sodium chloride (NaCl) solution (Sigma-Aldrich, St. Louis, MO, USA) were added, followed by incubation on a rocking platform at 4 °C for 1 h. The samples were centrifuged again under the same conditions, yielding a pellet containing viral particles. The pellet was resuspended in 500 µL of sterile phosphate-buffered saline (PBS, SIGMA, Merck Life Science, Madrid, Spain), homogenized, and transferred to sterile microtubes.

Nucleic acid extraction was performed from 150 µL of viral concentrate using the QIAamp Viral RNA Mini Kit (Qiagen, Hilden, Germany), according to the manufacturer’s instructions. The RNA was eluted in a final volume of 60 µL and either analyzed immediately or stored at −80 °C until reverse transcription quantitative polymerase chain reaction (RT-qPCR) analysis.

Three process controls were included in each analytical batch [29,30]. A sample process control (SPC) consisted of 40 mL of TGBE buffer spiked with the same amount of MgV and processed in parallel with the salad bowl samples. The SPC simulated an ideal recovery scenario and was used to verify the performance of the entire analytical procedure. A negative sample process control (NSPC) was included in all batches to detect potential cross-contamination during sample processing. This control was treated identically to the SPC but without MgV spiking. In addition, an extraction control (EC) was included in each batch to assess RNA extraction efficiency by spiking TGBE buffer with the same amount of MgV. MgV recovery was estimated using a comparative cycle threshold (Ct) approach previously applied for viral recovery assessment [29], as shown in Equation (1).

(1) $$Recovery\left(\%\right)=100\times {\;2}^{Ct\;SPC-Ct\;Sample}$$

*^Ct SPC^* represents the Ct value of process control, and *^Ct Sample^* represents the Ct value of MgV recovered from the corresponding sample. This approach assumes an amplification efficiency of approximately 100%. A minimum MgV recovery of 5% was adopted as a conservative quality-control criterion, which was more stringent than the ≥1% minimum recovery specified in ISO 15216-1:2017 [28]; recovery was classified as insufficient (<5%), acceptable (5–25%), good (25–50%), or very good (>50%), and samples with recovery values below 5% were considered unacceptable and reprocessed from the virus concentration step through nucleic acid extraction.

### 2.4. Detection of Enteric Viruses by RT-qPCR

MgV was quantified for recovery assessment, whereas NoV GI, NoV GII, HAstV, HAV, HEV, and RV were detected using one-step RT-qPCR assays. The final reaction volume was 10 µL and contained 2.5 µL of RNA, 0.2 µM of each primer, 0.2 µM of probe, and 1× TaqMan Fast Virus 1-Step Master Mix (Applied Biosystems, Waltham, MA, USA). Primer and probe sequences are presented in Table S3 [28,31,32,33].

Potential RT-qPCR inhibition was evaluated by comparing the results obtained from duplicate analyses of the undiluted RNA extract and the corresponding 1:10 dilution, with the diluted result adjusted for the dilution factor. Inhibition was considered acceptable when it did not exceed 75%, according to ISO 15216-1:2017 [28]. The SPC and EC were used to assess process and extraction performance for MgV, respectively, whereas the NSPC and assay-specific no-template controls were used to monitor potential contamination.

RT-qPCR assays were performed using a QuantStudio™ 5 Real-Time PCR System (Applied Biosystems, Waltham, MA, USA), and data were analyzed with QuantStudio™ Design & Analysis Software (v1.5.1) (Applied Biosystems, Waltham, MA, USA). Thermal cycling conditions were specific to each viral target and followed the corresponding reference assays; detailed cycling conditions are provided in Table S4.

For each viral target, a standard curve was included in every experiment using quantitative nucleic acid standard materials obtained from American Type Culture Collection (ATCC, Manassas, VA, USA) (Table S5) to monitor inter-assay performance and determine the limit of quantification (LOQ). Three-point standard curves were prepared using the initial standard concentration and two successive 10-fold dilutions. The concentrations were 13,750, 1375, and 137.5 genome copies per reaction for HAV, NoV GI, NoV GII, and HAstV; 550, 55, and 5.5 genome copies per reaction for HEV; and 2500, 250, and 25 genome copies per reaction for RV. Standard curve performance parameters, including slope, coefficient of determination (R^2^), and amplification efficiency, obtained from two independent RT-qPCR runs for each viral target are summarized in Table S6. For HEV, the LOQ was estimated as the lowest RNA standard concentration presenting a coefficient of variation below 35% between duplicate measurements [34], corresponding to 5.5 genome copies per reaction.

Samples were considered positive when a characteristic sigmoidal amplification curve was observed in both duplicate reactions, with a Ct value ≤ 40, and all negative and no-template controls showed no amplification. All samples, including both positive and negative samples, were analyzed in two independent RT-qPCR runs for each viral target using the same RNA extract, with each sample analyzed in technical duplicate in each run.

To ensure consistency across comparative analyses, the fluorescence threshold was manually standardized rather than relying on the software-generated value, which was calculated as 10 times the standard deviation of the baseline fluorescence [29,32]. Target-dependent thresholds were established from representative amplification curves by positioning the threshold within the early portion (approximately the first third) of the exponential amplification phase. Based on this criterion, the fluorescence threshold used for Ct determination was set at a ΔRn (baseline-corrected normalized reporter fluorescence) value of 0.04 for all enteric viruses except HAV (0.08), and at 0.07 for MgV.

For further molecular characterization of HEV-positive samples, first-strand complementary DNA (cDNA) was synthesized using the Transcriptor First Strand cDNA Synthesis Kit (Roche Diagnostics, Mannheim, Germany), following the manufacturer’s Procedure B. Briefly, cDNA synthesis was performed in a final volume of 20 µL using a combination of anchored-oligo(dT)18 and random hexamer primers. Reverse transcription was carried out at 25 °C for 10 min followed by 55 °C for 30 min, and the reverse transcriptase was subsequently inactivated at 85 °C for 5 min. The resulting cDNA was then used for partial HEV genome amplification.

### 2.5. Statistical Analysis

Physicochemical variables included pH, temperature and water activity (a_w_). Descriptive statistics, including mean, standard deviation, median, minimum and maximum values, were calculated for each salad type, sampling period and salad type × sampling period combination.

To avoid pseudoreplication, the dataset was organized with one row per independent salad sample. Each sample was assigned a unique identifier according to salad type and sampling period.

Differences in physicochemical characteristics were evaluated using two-way analysis of variance (ANOVA), with salad type, sampling period and their interaction as explanatory terms. Model assumptions were assessed using the Shapiro–Wilk test for residual normality, Levene’s test for homogeneity of variances and inspection of studentized residuals for extreme values. When a model assumption was not fully met, a permutation-based ANOVA sensitivity analysis was performed. When appropriate, pairwise comparisons were conducted using Tukey’s honestly significant difference (HSD) test with correction for multiple comparisons.

HEV RT-qPCR positivity was calculated using 30 independent samples as the denominator and was reported as the number and percentage of positive samples. Exact binomial 95% confidence intervals were calculated when appropriate. Associations between HEV detection and salad type were evaluated using the Fisher–Freeman–Halton exact test, whereas the association with sampling period was evaluated using Fisher’s exact test. Exploratory differences in pH, temperature, and water activity between HEV-positive and HEV-negative samples were evaluated using Mann–Whitney U tests.

As an exploratory analysis, Principal Component Analysis (PCA) was conducted using standardized pH, temperature, and water activity measurements to visualize the multivariate physicochemical structure of the samples. PCA was not used to evaluate associations with HEV detection. The corresponding analysis and biplot are provided in the Supplementary Materials.

Graphical exploratory analyses were performed to visualize the distribution of physicochemical parameters and HEV RT-qPCR positivity among salad types.

All statistical analyses were performed using R software v4.5.3 (R Foundation for Statistical Computing, Vienna, Austria). Statistical significance was established at *p* < 0.05.

## 3. Results

### 3.1. Physicochemical Characteristics of the Salad Samples

**Figure 1:**
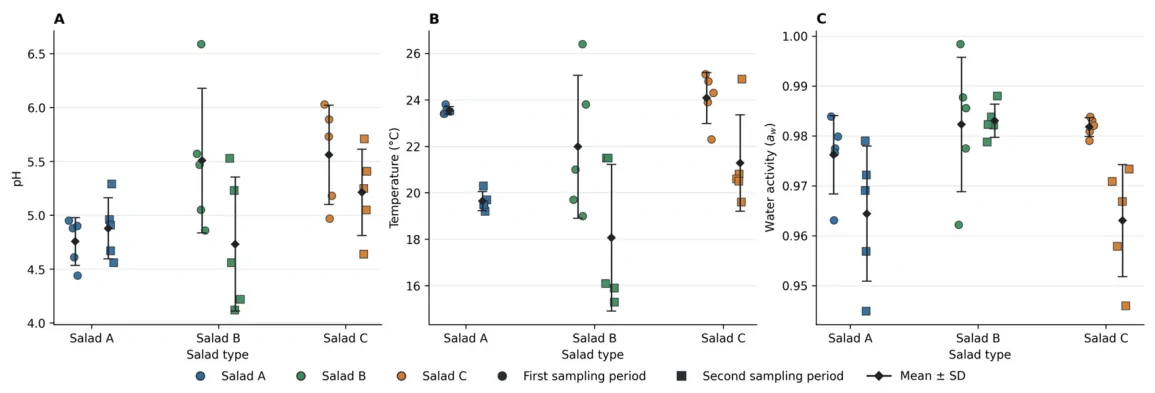
Physicochemical characteristics of the salad samples. (**A**) pH, (**B**) temperature, and (**C**) water activity. Each point represents one independent sample (*n* = 10 per salad type; five per sampling period). Horizontal lines and error bars represent the mean and standard deviation, respectively. Two-way ANOVA identified significant overall effects of salad type on pH, temperature, and water activity; however, Tukey-adjusted pairwise comparisons did not identify significant differences between individual types.

A total of 30 independent RTE salad samples were analyzed. Across all samples, pH ranged from 4.12 to 6.59, temperature ranged from 15.30 to 26.40 °C, and water activity ranged from 0.9449 to 0.9984 (Figure 1).

Model diagnostics indicated no extreme studentized residuals (|studentized residual| > 3), and the assumptions of residual normality and homogeneity of variance were met. A significant main effect of salad type on pH was observed (F = 3.62, *p* = 0.042). In contrast, neither the main effect of sampling period (F = 3.74, *p* = 0.065) nor the salad type × sampling period interaction (F = 2.26, *p* = 0.127) was statistically significant.

For temperature, residual normality was not met (Shapiro–Wilk, *p* = 0.028), whereas homogeneity of variances was not rejected (Levene’s test, *p* = 0.147). The conventional two-way ANOVA identified significant main effects of salad type (F = 4.27, *p* = 0.026) and sampling period (F = 22.44, *p* < 0.001). In contrast, the salad type × sampling period interaction was not significant (F = 0.25, *p* = 0.784). Given the deviation from residual normality, a permutation-based sensitivity analysis was performed, which confirmed the same pattern: significant effects of salad type (permutation *p* = 0.027) and sampling period (permutation *p* < 0.001), but no significant interaction (permutation *p* = 0.797).

For water activity, significant main effects were detected for salad type (F = 4.62, *p* = 0.020) and sampling period (F = 7.84, *p* = 0.010), whereas the salad type × sampling period interaction was not statistically significant (F = 2.59, *p* = 0.096).

Despite the significant overall effects of salad type, Tukey HSD post hoc comparisons did not identify statistically significant pairwise differences among salad types after adjustment for multiple comparisons. Regarding the sampling period, the second sampling period had a significantly lower temperature than the first (mean difference = −3.54 °C, adjusted *p* < 0.001) and lower water activity (mean difference = −0.0099, adjusted *p* = 0.024).

An exploratory PCA of the physicochemical variables further indicated substantial overlap among salad types and sampling periods (Figure S1).

### 3.2. Enteric Virus Occurrence and Associations with Physicochemical Variables

**Figure 2:**
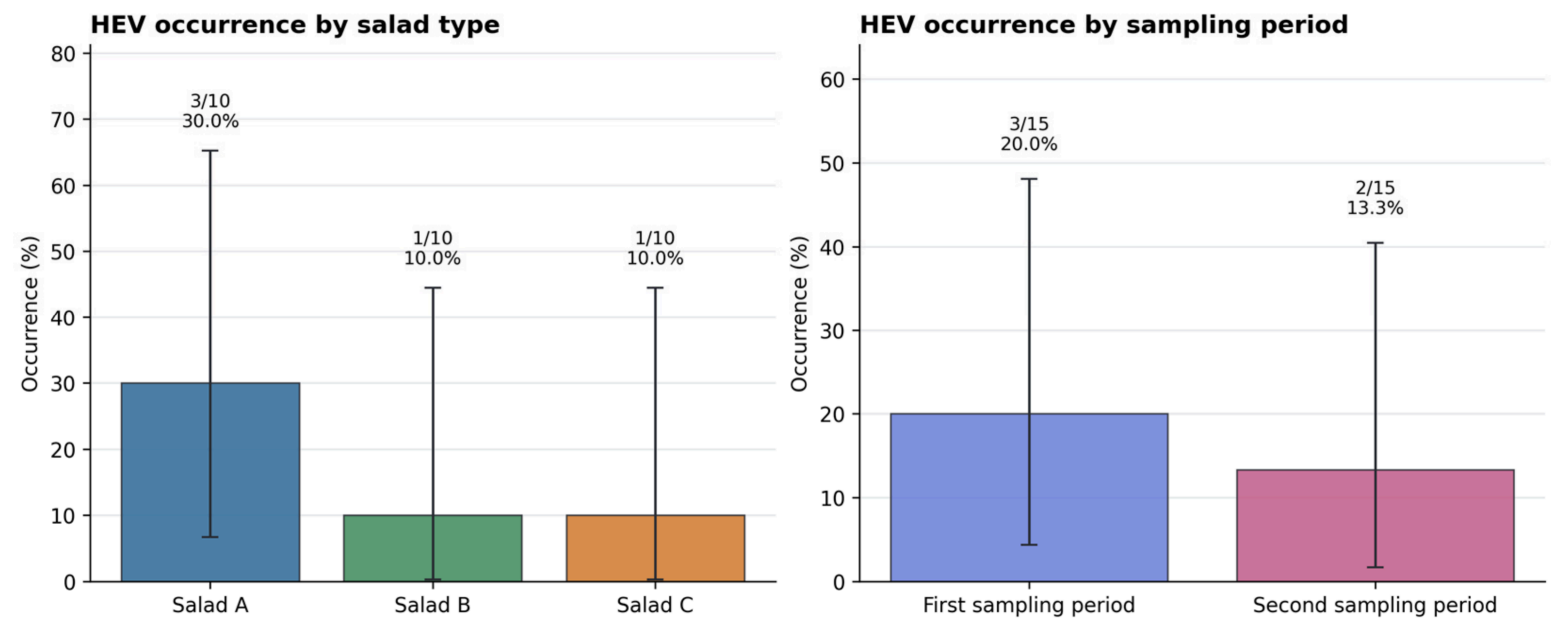
HEV RT-qPCR positivity in multi-component salad bowls obtained through last-mile delivery, according to salad type and sampling period. Bars represent the percentage of HEV RT-qPCR-positive samples, and error bars indicate exact binomial 95% confidence intervals. Values above the bars indicate the number of RT-qPCR-positive samples over the total number analyzed and the corresponding percentage.

The mean control virus recovery was 87.6%. Overall, 36.7% of samples showed acceptable recovery (5–25%), 10.0% good recovery (25–50%), and 53.3% very good recovery (>50%). Estimated RT-qPCR inhibition ranged from 0 to 73.8%, and none of the samples exceeded the 75% acceptance threshold [28]. HEV-target RT-qPCR signals were observed in 5 of 30 independent samples, corresponding to an overall RT-qPCR positivity of 16.7% (95% confidence interval [CI]: 5.6–34.7%). By salad type, HEV RT-qPCR positivity was observed in 3/10 Salad A samples (30.0%), 1/10 Salad B samples (10.0%), and 1/10 Salad C samples (10.0%). By sampling period, HEV RT-qPCR positivity was observed in 3/15 samples from the first sampling period (20.0%) and 2/15 samples from the second sampling period (13.3%) (Figure 2). Although none of the HEV-positive salad formulations contained pork, pork-containing dishes were identified on the online menus of all three establishments.

Only 5 positive events were available among 30 independent samples. A full multivariable logistic regression model including salad type, sampling period, pH, temperature, and water activity would require more parameters than supported by the number of events. Therefore, a conventional multivariable logistic regression model was not fitted for inferential purposes. Exploratory univariable analyses did not detect statistically significant associations between HEV detection and the evaluated categorical or physicochemical predictors. Fisher’s exact testing did not detect a statistically significant association between HEV detection and salad type (Fisher–Freeman–Halton exact *p* = 0.574) or sampling period (Fisher’s exact *p* = 1.000). Likewise, no significant differences were observed between HEV-positive and HEV-negative samples for pH (Mann–Whitney U = 33.5, *p* = 0.113), temperature (U = 71.0, *p* = 0.656), or water activity (U = 59.5, *p* = 0.889). HEV-positive samples had a median pH of 4.56 (range: 4.12–5.73), compared with 5.05 (4.22–6.59) among negative samples; a median temperature of 22.30 °C (20.30–23.50 °C), compared with 20.80 °C (15.30–26.40 °C); and a median water activity of 0.98 (0.96–0.98), compared with 0.98 (0.94–1.00).

All samples were analyzed in two independent RT-qPCR runs for each viral target. HEV-target amplification was reproducibly observed in both technical duplicates in both runs for the same five samples, whereas no amplification was observed for NoV GI, NoV GII, HAV, HAstV, or RV in either run. Positive samples had mean Ct values ranging from 37.97 to 39.48, and all values were below the estimated LOQ of 5.5 genome copies per reaction. All five HEV-positive signals were detected in the undiluted RNA extracts, whereas no HEV-target amplification was observed in the corresponding 1:10 dilutions. Overall, low-level HEV-target RT-qPCR positivity was observed sporadically across the analyzed RTE salad products, and no statistically significant association with salad type, sampling period, pH, temperature, or water activity was identified within the analyzed dataset (Table 1).

To characterize the HEV strains, the five RNA-positive samples underwent partial genome amplification. However, none of the samples yielded amplicons of sufficient quality or concentration for sequencing. The unsuccessful amplification may have been related to the low amount of HEV RNA in the samples, as indicated by their high Ct values.

## 4. Discussion

This study investigated the occurrence of multiple enteric viruses in multi-component RTE salad bowls obtained through a last-mile food delivery system. HEV-target RT-qPCR signals were observed in 16.7% of the samples analyzed, whereas no RT-qPCR amplification was observed for NoV GI, NoV GII, HAV, HAstV, or RV. The HEV RT-qPCR-positive samples had high Ct values, ranging from 37.97 to 39.48, and all signals were below the estimated LOQ. No statistically significant association was observed between HEV RT-qPCR status and salad type, sampling period, or the measured physicochemical characteristics. Despite the low RNA concentrations, it is important to note that only a 25 g portion of each salad was analyzed. Therefore, consumption of an entire bowl could result in greater overall exposure if viral contamination were distributed throughout the product. However, because all HEV-positive signals were below the LOQ and MgV recovery was used as a process-control measure rather than to correct HEV concentrations, the total viral load per serving could not be reliably estimated.

Multi-component salad bowls combine ingredients with distinct production histories and may undergo several handling and preparation steps before consumption. Consequently, contamination may have occurred upstream during primary production or processing, during meal preparation and handling, or at another point before the product reached the destination [18,35], with last-mile delivery representing the distribution context through which the products were obtained and evaluated rather than an identified source of contamination.

Although the source of contamination could not be clearly established, some plausible hypotheses can be considered. As extensively reported in the literature, pigs are the main reservoir of zoonotic HEV, especially genotypes 3 and 4, and an important source of foodborne transmission [36]. Only one of the analyzed salad formulations contained ham (samples B1.4 and B2.4), and neither sample was HEV-positive. Nevertheless, a review of the restaurants’ menus indicated that all establishments offered pork-containing dishes. Therefore, cross-contamination during food preparation or handling cannot be excluded as a potential route of contamination.

The animal-derived ingredients included in the salad bowls were salmon, tuna, chicken, and prawns, for which there is currently limited evidence supporting their role as established sources of human-pathogenic HEV. Consequently, the contamination may instead have originated from vegetable ingredients, which can become contaminated during primary production through exposure to fecally contaminated irrigation water, representing a recognized route for the introduction of enteric viruses into the fresh produce supply chain [37].

Published evidence specifically investigating the occurrence of enteric viruses in foods obtained through last-mile delivery systems appears to be limited. Previous studies have reported the presence of enteric viruses in RTE foods. Terio et al. (2017) detected HAV and HEV in 1.9% (18/911) and 0.6% (6/911) of RTE vegetable samples in Italy, respectively [8]. In contrast, NoV GI and NoV GII were not detected in any of the samples analyzed [8]. Kokkinos et al. (2012) investigated the occurrence of human enteric viruses, including HAV, HEV, NoV GI, and NoV GII, throughout the leafy green vegetable supply chain in three European countries [38]. HAV RNA was detected in 1.32% (4/304) of samples, HEV RNA in 3.42% (5/146), NoV GI RNA in 2.0% (6/299), and NoV GII RNA in 2.95% (8/271). Among the positive detections, none of the HAV-positive samples, four of the HEV-positive samples, two of the NoV GI-positive samples, and one of the NoV GII-positive samples were food samples, specifically fresh lettuce. All remaining positive detections occurred in non-food samples collected along the supply chain [38].

The studies discussed above provide further context for the non-detection of NoV GI, NoV GII, HAV, HAstV, and RV in the present study. Similarly, other investigations have reported variable detection patterns for enteric viruses in RTE and salad vegetables. Mattison et al. (2010) detected NoV RNA in 6% of RTE packaged leafy green samples collected from retail markets in Canada, whereas RV was detected in only 0.4% of samples [23]. Similarly, Cook et al. (2019) detected NoV in 5.3% (30/568) of lettuce samples sold at retail in the United Kingdom [39]. In contrast, Terio et al. (2020) reported a considerably higher occurrence of NoV GII RNA, detecting the virus in 74.8% of 135 RTE salad samples collected from two vegetable-processing plants [25]. More recently, Neetoo et al. (2023) also reported the presence of NoV in salad vegetables, with detection rates varying according to the food matrix, including 36% in lettuce, 16% in watercress, and 4% in tomatoes [9]. Taken together, these findings highlight the marked variability in the occurrence of enteric viruses across studies, food matrices, geographical settings, and sampling schemes. Therefore, the non-detection of NoV GI, NoV GII, HAV, HAstV, and RV in the present study is not inconsistent with the existing literature. Rather, it may reflect the sporadic and heterogeneous viral contamination in RTE vegetables and multi-component foods.

Regarding the physicochemical analysis, salad type had a significant overall effect on pH, temperature, and water activity, although Tukey HSD post hoc comparisons did not identify significant pairwise differences among individual salad types after adjustment for multiple comparisons. Sampling period significantly affected temperature and water activity, with samples from the second sampling period showing lower values for both variables than those from the first sampling period, whereas no significant salad type × sampling period interactions were observed. No evaluated physicochemical variable was significantly associated with HEV RNA detection. These parameters characterize the microenvironment of the food matrix at the time the product reaches the consumer and may, in principle, influence the persistence and stability of viruses already present in the food, as demonstrated in previous studies [40,41,42,43], although they do not determine the initial occurrence of contamination. However, because RNA detection does not provide information on viral infectivity, it was not possible to assess whether the physicochemical conditions observed in the present study influenced HEV infectivity or persistence.

The study has some limitations that should be acknowledged. First, the detection of HEV-target RT-qPCR signals does not provide direct evidence of the presence of infectious viruses. Therefore, although HEV-target RT-qPCR signals were detected in the food matrix analyzed, these findings do not establish whether infectious virus was present or allow the associated consumer risk to be determined. Nevertheless, molecular detection remains relevant for food safety surveillance, as the presence of viral RNA provides evidence that viral material has entered the food chain and can help identify contaminated food matrices, potential contamination routes, and areas requiring further investigation. This is particularly relevant for emerging foodborne viruses such as HEV, for which occurrence data remain limited in many food matrices. International food-safety bodies recognize nucleic-acid-based methods as important tools for the detection and monitoring of foodborne viruses, while acknowledging their inability to distinguish infectious from non-infectious virus particles [44,45].

Secondly, the high Ct values indicated very low viral RNA concentrations below the quantitative range of the assay, where stochastic variability increases and quantitative precision decreases; these low RNA levels prevented successful molecular characterization and precluded comparison with potential human, animal, or environmental sources. The absence of HEV-target amplification in the corresponding 1:10 dilutions is consistent with the very low target concentrations indicated by the high Ct values observed in the undiluted extracts. Although dilution can mitigate RT-qPCR inhibition, when present, at such low target concentrations, a ten-fold dilution may further reduce the number of target RNA molecules available for amplification, thereby decreasing the probability of detection. Similar loss of detection after 1:10 dilution has been reported for low-level viral RNA in food matrices [46,47]. Therefore, the absence of amplification in the diluted extracts is consistent with the very low-level nature of the HEV-target signals and further reinforces the need for cautious interpretation.

The public health significance of low-level HEV RNA detection should be interpreted cautiously [45,48]. The oral infectious dose of HEV in humans has not been established [48]. Experimental studies in pigs indicate a dose-dependent probability of infection, with oral infection reported at approximately 10^6^ genomic copies, although these estimates cannot be directly extrapolated to humans because infectivity depends on factors such as host susceptibility, viral strain, exposure route, and food matrix [49]. Moreover, the number of HEV genome copies detected by RT-qPCR cannot be directly translated into a corresponding number of infectious particles [45,50]. Approaches such as viability RT-qPCR using intercalating compounds, capsid- or genome-integrity assays, and cell culture have been explored to better assess HEV infectivity. However, molecular viability assays provide only indirect proxies of infectivity and may overestimate infectious viruses, whereas HEV cell culture remains technically challenging and standardized infectivity methods for food samples are still limited [50,51].

Thirdly, the geographical origin of the individual ingredients was not available and could therefore not be considered when interpreting the HEV findings. Finally, the limited number of samples analyzed should be considered when interpreting the findings, as the absence of detection within this dataset does not demonstrate the broader absence of the viruses investigated from multi-component RTE salad bowls. For multi-component RTE salad bowls, differences in ingredients, ingredient sourcing, food preparation and handling practices, local contamination patterns, seasonal conditions, and distribution pathways may influence viral occurrence. Previous occurrence studies of enteric viruses in fresh and RTE produce have also been conducted within geographically and temporally defined sampling frameworks, including specific food categories, retail settings, or production facilities. For HEV, specifically, Di Cola et al. reported no positive samples among the analyzed leafy green vegetables and berries, whereas Terio et al. (2017) detected HEV RNA in only 0.6% (6/911) of RTE vegetable samples [8,10], as mentioned before. Other studies have reported HEV RNA in 3.42% (5/146) of samples from the leafy green vegetable supply chain [38], and in 1.4% of vegetable samples collected in Sicily [52]. These previously reported HEV RT-qPCR positivity rates were lower than those observed in the present study (16.7%, 5/30). Such variability may reflect differences in geographical setting, sampling period, food matrix, production and handling practices, analytical methodology, and sampling design. Therefore, the HEV occurrence observed in the present study should be interpreted as specific to the products and sampling framework evaluated and should not be considered representative of other geographical regions, periods of the year, food categories, or delivery systems.

## 5. Conclusions

The present study assessed multi-component RTE salad bowls obtained through a third-party delivery platform and analyzed as received at the consumer-facing endpoint of the food chain, thereby capturing the final product after preparation and last-mile distribution. HEV-target RT-qPCR signals were observed in 16.7% of the samples, whereas no RT-qPCR amplification was observed for NoV GI, NoV GII, HAV, HAstV, or RV. No statistically significant association was observed between HEV RT-qPCR status and salad type, sampling period, or the measured physicochemical characteristics.

The findings demonstrate that HEV RT-qPCR signals can be present in complex RTE meals as delivered to consumers, while also highlighting the challenges of identifying the contamination source and interpreting the public health significance of low-level viral RNA detection in multi-component foods. Rather than identifying last-mile delivery as a source of contamination, the study supports the inclusion of emerging food distribution pathways in foodborne virus surveillance and highlights the need for broader investigations to better characterize contamination routes, viral infectivity, and potential consumer risk.

## Figures and Tables

**Table 1:** Multi-component salad bowl samples obtained through last-mile delivery showing HEV-target RT-qPCR positivity and corresponding Ct values.

Sample ID	Salad Type	Sampling Period	Mean Ct Values	MgV Recovery (%)	Recovery Classification
A1.2	Salad A	First sampling period	39.14	13.6	Acceptable
A1.4			39.04	19.7	Acceptable
A2.2		Second sampling period	39.48	169.6	Very good
B2.2	Salad B	Second sampling period	37.97	151.4	Very good
C1.1	Salad C	First sampling period	39.23	88.7	Very good
Number of positive samples			Ct value, median (min–max)		
5			39.14 (37.97–39.48)		

Mean Ct values were calculated from two independent RT-qPCR runs; each performed in technical duplicate. HEV-target amplification was reproducibly observed in both technical duplicates in both independent runs. All signals were below the estimated LOQ; therefore, reliable concentrations in genome copies per gram could not be determined.

## Data Availability

The raw data supporting the findings of this article will be made available by the authors upon reasonable request.

## Supplementary Materials

The following supporting information can be downloaded at https://www.mdpi.com/article/10.3390/v18091000/s1, Figure S1: Principal component analysis (PCA) biplot of the physicochemical characteristics of ready-to-eat salad samples; Table S1: Ingredient composition of the ready-to-eat salad bowls analyzed during the two sampling periods; Table S2: Sample-specific delivery conditions, packaging, weight, and core temperature upon arrival at the laboratory; Table S3: Oligonucleotides and TaqMan™ probes for enteric viruses and MgV used in the study; Table S4: Thermal cycling conditions used for the RT-qPCR detection of enteric viruses; Table S5: Exogenous standard materials obtained from ATCC; Table S6: Standard curve performance parameters for each viral target across two independent RT-qPCR runs. Refs. [28,31,32,33] are cited in Supplementary Materials.

## Abbreviations

The following abbreviations are used in this manuscript:

ANOVA	Analysis of variance
ATCC	American Type Culture Collection
a_w_	Water activity
CAPES	Coordination for the Improvement of Higher Education Personnel
CECT	Spanish Type Culture Collection
CI	Confidence interval
COVID-19	Coronavirus disease 2019
Ct	Cycle threshold
EC	Extraction control
EFSA	European Food Safety Authority
ENZOEM	Zoonoses and Emerging Diseases
FAO	Food and Agriculture Organization of the United Nations
HAstV	Human astrovirus
HAV	Hepatitis A virus
HCl	Hydrochloric acid
HEV	Hepatitis E virus
hAdV	Human adenovirus
HSD	Honestly significant difference
IU	Infective units
ISO	International Organization for Standardization
LOQ	Limit of quantification
MgV	Mengovirus
NaCl	Sodium chloride
NoV	Norovirus
NoV GI	Norovirus genogroup I
NoV GII	Norovirus genogroup II
NSPC	Negative sample process control
OED	Oxford English Dictionary
PBS	Phosphate-buffered saline
PC1	First principal component
PC2	Second principal component
PCA	Principal component analysis
PEG	Polyethylene glycol
RNA	Ribonucleic acid
RT-qPCR	Reverse transcription quantitative polymerase chain reaction
RTE	Ready-to-eat
RV	Rotavirus
SPC	Sample process control
TGBE	Tris-glycine-beef extract
UIC	Competitive Research Unit
WHO	World Health Organization
