# Supplementary material for: Occurrence of Enteric Viruses in Multi-Component Salad Bowls
Source: Viruses. 2026 Sep 11;18(9):1000. doi: 10.3390/v18091000 (PMC13612071; doi:10.3390/v18091000)

Supplementary Material

Figure S1: Principal component analysis (PCA) biplot of the physicochemical characteristics of ready-to-eat salad samples. The analysis was performed using pH, temperature, and water activity (a_w) measurements. Each point represents an independent sample, with colors indicating salad types A, B, and C, whereas point shapes indicate the first and second sampling periods. Arrows represent the loadings of the physicochemical variables, with their direction indicating the association with the principal components and their length reflecting their relative contribution to sample differentiation.

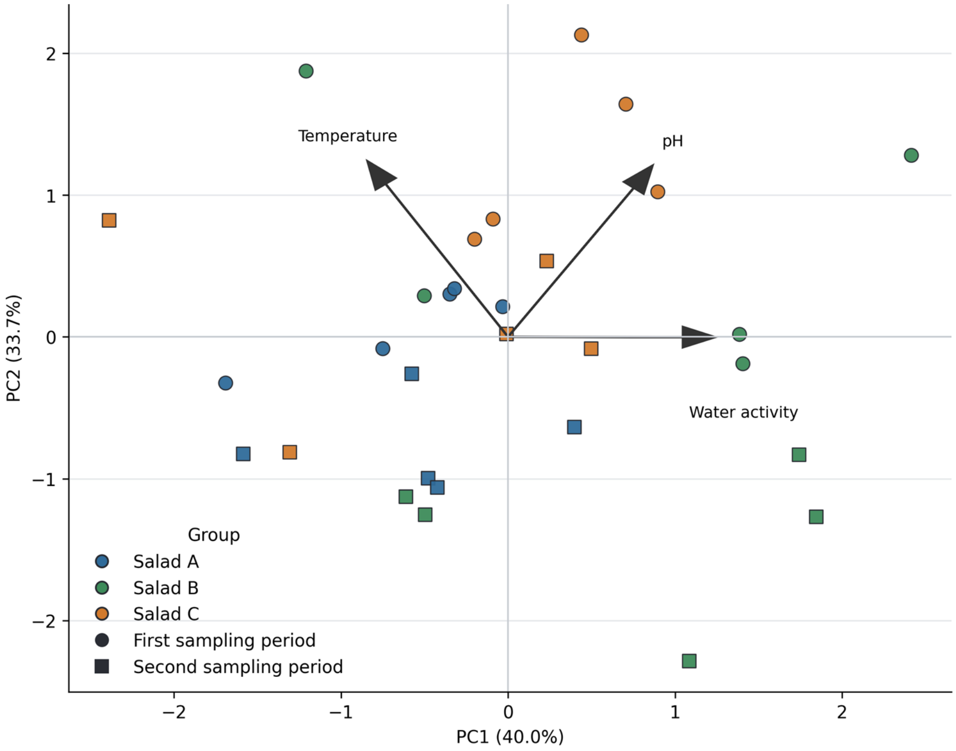

Supplement: Supplementary file 1 [file viruses-18-01000-s001.zip › Supplementary Material_Figures.pdf]
