# Supplementary material for: Occurrence of Enteric Viruses in Multi-Component Salad Bowls
Source: Viruses. 2026 Sep 11;18(9):1000. doi: 10.3390/v18091000 (PMC13612071; doi:10.3390/v18091000)
Supplement: Supplementary file 1 [file viruses-18-01000-s001.zip › Supplementary Material_Tables.pdf]

Supplementary Material

Table S1: Ingredient composition of the ready-to-eat salad bowls analyzed during the two sampling periods.

Salad type	First sampling period	Second sampling period	Ingredients
Salad A	A1.1	A2.1	Sushi rice, salmon, sauce (ingredients not specified), edamame, cucumber, avocado, carrot, and sesame seeds
	A1.2	A2.2	Sushi rice, tuna, red sauce, pineapple, cucumber, jalapeños, cherry tomatoes, bean sprouts, and crispy onion
	A1.3	A2.3	Sushi rice, quinoa, chicken, teriyaki sauce, jalapeños, wakame seaweed, mango, carrot, and spring onion
	A1.4	A2.4	Sushi rice, prawns, sweet chilli sauce, wakame seaweed, red onion, pineapple, sweetcorn, jalapeño, and spring onion
	A1.5	A2.5	Sushi rice, tuna, salmon, mayonnaise sauce, bean sprouts, red onion, pineapple, mango, and spring onion

Salad B	B1.1	B2.1	Mixed green leaves, tomato, onion, sweetcorn, olives, carrot, tuna, and egg
	B1.2	B2.2	Mixed green leaves, tomato, onion, olives, sun-dried tomatoes, feta cheese, and chicken
	B1.3	B2.3	Mixed green leaves, tomato, onion, chicken, olives, Parmesan cheese, Caesar dressing, and croutons
	B1.4	B2.4	Mixed green leaves, tomato, onion, ham, feta cheese, Neapolitan sauce, egg, pasta, and rocket
	B1.5	B2.5	Mixed green leaves, burrata cheese, tomato, sun-dried tomatoes, avocado, balsamic vinegar, walnuts, and rocket
Salad C	C1.1	C2.1	Gluten-free sushi rice base with diced salmon and bluefin tuna, wakame seaweed, edamame, mango, cucumber, fried onion, sauce (ingredients not specified), and teriyaki sauce
	C1.2	C2.2	Gluten-free sushi rice base with diced bluefin tuna, wakame seaweed, edamame, mango, cucumber, fried onion, sauce (ingredients not specified), and teriyaki sauce

C1.3	C2.3	Gluten-free sushi rice base with teriyaki chicken, wakame seaweed, edamame, mango, cucumber, fried onion, sauce (ingredients not specified), and teriyaki sauce
C1.4	C2.4	Gluten-free sushi rice base with diced salmon, wakame seaweed, edamame, mango, cucumber, fried onion, sauce (ingredients not specified), and teriyaki sauce
C1.5	C2.5	Wakame seaweed, cherry tomatoes, tofu, pumpkin, and peanuts

Table S2: Sample-specific delivery conditions, packaging, weight, and core temperature upon arrival at the laboratory.

Sample ID	Sampling period	Transport mode and condition	Transport time	Packaging	Sample weight (g)	Core temperature upon arrival (°C)
A1.1	First	Third-party delivery platform – bicycle, non-refrigerated	10–20 min	Cardboard bowl with plastic lid	621	23.4
A1.2	First	Third-party delivery platform – bicycle, non-refrigerated	10–20 min	Cardboard bowl with plastic lid	632	23.4
A1.3	First	Third-party delivery platform – bicycle, non-refrigerated	10–20 min	Cardboard bowl with plastic lid	600	23.8
A1.4	First	Third-party delivery platform – bicycle, non-refrigerated	10–20 min	Cardboard bowl with plastic lid	616	23.5
A1.5	First	Third-party delivery platform – bicycle, non-refrigerated	10–20 min	Cardboard bowl with plastic lid	628	23.6
B1.1	First	Third-party delivery platform – bicycle, non-refrigerated	10–20 min	Cardboard bowl with plastic lid	406.7	19.7
B1.2	First	Third-party delivery platform – bicycle, non-refrigerated	10–20 min	Cardboard bowl with plastic lid	352	23.8
B1.3	First	Third-party delivery platform – bicycle, non-refrigerated	10–20 min	Cardboard bowl with plastic lid	470	26.4

B1.4	First	Third-party delivery platform – bicycle, non-refrigerated	10–20 min	Cardboard bowl with plastic lid	451	19.0
B1.5	First	Third-party delivery platform – bicycle, non-refrigerated	10–20 min	Cardboard bowl with plastic lid	463	21.0
C1.1	First	Third-party delivery platform – bicycle, non-refrigerated	10–20 min	Cardboard bowl with plastic lid	511	22.3
C1.2	First	Third-party delivery platform – bicycle, non-refrigerated	10–20 min	Cardboard bowl with plastic lid	522	24.3
C1.3	First	Third-party delivery platform – bicycle, non-refrigerated	10–20 min	Cardboard bowl with plastic lid	537	24.8
C1.4	First	Third-party delivery platform – bicycle, non-refrigerated	10–20 min	Cardboard bowl with plastic lid	509	23.9
C1.5	First	Third-party delivery platform – bicycle, non-refrigerated	10–20 min	Cardboard bowl with plastic lid	512	25.1
A2.1	Second	Third-party delivery platform – bicycle, non-refrigerated	10–15 min	Cardboard bowl with plastic lid	619	19.7
A2.2	Second	Third-party delivery platform – bicycle, non-refrigerated	10–15 min	Cardboard bowl with plastic lid	571	20.3
A2.3	Second	Third-party delivery platform – bicycle, non-refrigerated	10–15 min	Cardboard bowl with plastic lid	633	19.5

A2.4	Second	Third-party delivery platform – bicycle, non-refrigerated	10–15 min	Cardboard bowl with plastic lid	635	19.2
A2.5	Second	Third-party delivery platform – bicycle, non-refrigerated	10–15 min	Cardboard bowl with plastic lid	642	19.5
B2.1	Second	Company car, non-refrigerated	20–30 min	Cardboard bowl with plastic lid	405.8	16.1
B2.2	Second	Company car, non-refrigerated	20–30 min	Cardboard bowl with plastic lid	319.2	21.5
B2.3	Second	Company car, non-refrigerated	20–30 min	Cardboard bowl with plastic lid	360.7	21.5
B2.4	Second	Company car, non-refrigerated	20–30 min	Cardboard bowl with plastic lid	450.9	15.9
B2.5	Second	Company car, non-refrigerated	20–30 min	Cardboard bowl with plastic lid	471	15.3
C2.1	Second	Third-party delivery platform – bicycle, non-refrigerated	10–15 min	Cardboard bowl with plastic lid	565	20.8
C2.2	Second	Third-party delivery platform – bicycle, non-refrigerated	10–15 min	Cardboard bowl with plastic lid	503	20.6
C2.3	Second	Third-party delivery platform – bicycle, non-refrigerated	10–15 min	Cardboard bowl with plastic lid	529	24.9

C2.4	Second	Third-party delivery platform – bicycle, non-refrigerated	10–15 min	Cardboard bowl with plastic lid	543	19.6
C2.5	Second	Third-party delivery platform – bicycle, non-refrigerated	10–15 min	Cardboard bowl with plastic lid	496	20.5

Table S3: Oligonucleotides and TaqMan™ probes for enteric viruses and MgV used in the study.

Viruses	Primers	Primer sequences	References
HEV	HEVj_FW	GGT GGT TTC TGG GGT GAC	[31]
	HEVj_RV	AGG GGT TGG TTG GAT GAA	
	HEVj_P	FAM-TGA TTC TCA GCC CTT CGC -BHQ1	
HAV	HAV68	TCA CCG CCG TTT GCC TAG	[28]
	HAV240	GGA GAG CCC TGG AAG AAA G	
	HAV150	FAM-CCT GAA CCT GCA GGA ATT AA-MGB	
HAstV	AstVorf1b+	AAG CAG CTT CGT GAC TCT GG	[33]
	AstVorf1b-	AGC CAT CAC ACT TCT TTG GTC	
	AstVorf1bp	FAM-AGA GCA ACT CCA TCG CAT TT-BHQ1	
RV	JVKF	CAG TGG TTG ATG CTC AAG ATG GA	[32]
	JVKR	TCA TTG TAA TCA TAT TGA ATA CCC A	

	JVKP	FAM-ACA ACT GCA GCT TCA AAA GAA GWG T- BHQ1	
NoV GI	QNIF4	CGC TGG ATG CGN TTC CAT	[28]
	NV1LCR	CCT TAG ACG CCA TCA TCA TTT AC	
	NVGG1p	FAM-TGG ACA GGA GAY CGC RAT CT-BHQ1	
NoV GII	QNIF2	ATG TTC AGR TGG ATG AGR TTC TCW GA	[28]
	COG2R	TCG ACG CCA TCT TCA TTC ACA	
	QNIFs	FAM-AGC ACG TGG GAG GGC GAT CG-BHQ1	
MgV	Mengo 110	GCG GGT CCT GCC GAA AGT	[28]
	Mengo 209	GAA GTA ACA TAT AGA CAG ACG CAC AC	
	Mengo 147	FAM-ATC ACA TTA CTG GCC GAA GCMGB-BHQ1	

Table S4: Thermal cycling conditions used for the RT-qPCR detection of enteric viruses.

Viral target	Reverse transcription	Initial denaturation	Amplification cycles	Reference
HEV	50 °C, 30 min	95 °C, 15 min	45 cycles: 95 °C, 10 s; 55 °C, 20 s; 72 °C, 15 s	[31]
HAV<br>NoV GI<br>NoV GII<br>MgV	55 °C, 60 min	95 °C, 5 min	45 cycles: 95 °C, 15 s; 60 °C, 1 min; 65 °C, 1 min	[28]
HAstV	55 °C, 60 min	95 °C, 5 min	45 cycles: 95 °C, 15 s; 58 °C, 1 min; 65 °C, 1 min	[33]
RV	50 °C, 30 min	95 °C, 15 min	45 cycles: 94 °C, 10 s; 55 °C, 30 s; 72 °C, 20 s	[32]

Table S5: Exogenous standard materials obtained from ATCC.

Virus	Reference
**NoV GI**	Quantitative Synthetic Norovirus G1 (I) RNA (ATCC VR-3234SD)
**NoV GII**	Quantitative Synthetic RNA from Norovirus G2 (II) (ATCC VR-3235SD)
**HAstV**	Quantitative Synthetic RNA from Astrovirus (ATCC VR-3238SD)
**HAV**	Quantitative Synthetic DNA from Hepatitis A virus (ATCC VR-3257SD)
**HEV**	Quantitative Synthetic RNA from Hepatitis E virus (ATCC VR-3258SD)
**RV**	Quantitative Genomic RNA from Rotavirus A strain Wa (TC adapted) (ATCC VR-2018DQ)

Table S6: Standard curve performance parameters for each viral target across two independent RT-qPCR runs.

Target	Slope, mean (range)	R^2, range	Efficiency, mean (range), %
HAV	-3.508 (-3.593 to -3.423)	0.9988–0.9988	92.9 (89.8–96.0)
HEV	-3.454 (-3.486 to -3.422)	0.9979–0.9988	94.8 (93.6–96.0)
NoV GI	-3.564 (-3.589 to -3.539)	0.9996–1.0000	90.9 (90.0–91.7)
NoV GII	-3.449 (-3.533 to -3.365)	0.9992–0.9994	95.1 (91.9–98.3)
HAstV	-3.415 (-3.462 to -3.368)	0.9982–0.9986	96.3 (94.5–98.1)
RV	-3.586 (-3.651 to -3.521)	0.99998–1.00000	90.1 (87.9–92.3)

Values represent the mean and range obtained from two independent RT-qPCR runs (n = 2) for each viral target.

References

28. ISO 15216 ISO 15216-1:2017 - Microbiology of the Food Chain — Horizontal Method for Determination of Hepatitis A Virus and Norovirus Using Real-Time RT-PCR — Part 1: Method for Quantification Available online: https://www.iso.org/standard/65681.html (accessed on 16 July 2026).

31. Jothikumar, N.; Cromeans, T.L.; Robertson, B.H.; Meng, X.J.; Hill, V.R. A Broadly Reactive One-Step Real-Time RT-PCR Assay for Rapid and Sensitive Detection of Hepatitis E Virus. *J. Virol. Methods* **2006**, *131*, 65–71, doi:10.1016/j.jviromet.2005.07.004.

32. Jothikumar, N.; Kang, G.; Hill, V.R. Broadly Reactive TaqMan® Assay for Real-Time RT-PCR Detection of Rotavirus in Clinical and Environmental Samples. *J. Virol. Methods* **2009**, *155*, 126–131, doi:10.1016/j.jviromet.2008.09.025.

33. Sano, D.; Pintó, R.M.; Omura, T.; Bosch, A. Detection of Oxidative Damages on Viral Capsid Protein for Evaluating Structural Integrity and Infectivity of Human Norovirus. *Environ. Sci. Technol.* **2010**, *44*, 808–812, doi:10.1021/es9018964.
